# Doublecortin-like kinase 1 promotes hepatocyte clonogenicity and oncogenic programming via non-canonical β-catenin-dependent mechanism

**DOI:** 10.1038/s41598-020-67401-y

**Published:** 2020-06-29

**Authors:** Naushad Ali, Charles B. Nguyen, Parthasarathy Chandrakesan, Roman F. Wolf, Dongfeng Qu, Randal May, Tatiana Goretsky, Javid Fazili, Terrence A. Barrett, Min Li, Mark M. Huycke, Michael S. Bronze, Courtney W. Houchen

**Affiliations:** 1 Department of Medicine, Section of Digestive Diseases and Nutrition, University of Oklahoma Health Sciences Center, Oklahoma City, OK 73104 USA; 2 Peggy and Charles Stephenson Cancer Center, University of Oklahoma Health Sciences Center, Oklahoma City, OK73104 USA; 3 Department of Veterans Affairs Medical Center, University of Oklahoma Health Sciences Center, Oklahoma City, OK 73104 USA; 4 Department of Radiation Oncology, University of Oklahoma Health Sciences Center, Oklahoma City, OK 73104 USA; 5 Division of Gastroenterology, Department of Internal Medicine, University of Kentucky, Lexington, KY 40536 USA

**Keywords:** Hepatocellular carcinoma, Diseases

## Abstract

Chronic liver injury is a risk factor for cirrhosis and hepatocellular carcinoma (HCC). The molecular mechanisms that regulate the decision between normal injury repair and neoplastic initiation are unclear. Doublecortin-like kinase 1 (DCLK1), a tumor stem cell marker, is induced during cirrhosis and HCC. Here, we demonstrate that DCLK1-overexpressing primary human hepatocytes formed spheroids in suspension cultures. Spheroids derived from DCLK1-overexpressing hepatoma cells showed high level expression of active β-catenin, α-fetoprotein, and SOX9, suggesting that DCLK1 overexpression induces clonogenicity and dedifferentiated phenotypes in hepatoma cells. DCLK1 overexpression in hepatoma cells also increased phosphorylation of GSK-3β at Ser^9^. This was associated with an induction of a 48-kDa active β-catenin with a preserved hypophosphorylated N-terminus that interacted with nuclear TCF-4 resulting in luciferase reporter activity and cyclin D1 expression. DCLK1 downregulation inhibited 48-kDa β-catenin expression. The proteasome inhibitor bortezomib did not block the 48-kDa β-catenin, instead, caused a threefold accumulation, suggesting a proteasome-independent mechanism. Liver tissues from patients with cirrhosis and HCC revealed epithelial co-staining of DCLK1 and active β-catenin, and cleaved E-cadherin. Repopulated DCLK1-overexpressing primary human hepatocytes in humanized FRG mouse livers demonstrated active β-catenin. In conclusion, DCLK1 regulates oncogenic signaling and clonogenicity of hepatocytes by a novel non-canonical/atypical β-catenin-dependent mechanism.

## Introduction

Hepatocellular carcinoma (HCC) is the second most common cause of cancer-related mortality and accounts for approximately 745,000 deaths per year worldwide^1^. Chronic liver injury due to hepatitis B/C viral infections (HBV and HCV), metabolic disorders, and alcohol are the major risk factors for HCC^2^. Recent reports using genetic lineage tracing models have demonstrated that mature hepatocytes are the cells of origin in injury-induced HCC^3^. Tumorigenesis in the liver is attributable to hepatocyte plasticity, defined as the ability of the mature hepatocytes to undergo phenotype change usually by dedifferentiation or transdifferentiation into a preneoplastic precursor cell-type^4–6^. The existing paradigm that hepatic facultative stem/progenitor cells are activated to produce different cell types during liver injury has been challenged in favor of hepatocyte plasticity^5,7,8^. This intuitive concept rests upon observations that mature hepatocytes retain the capacity to undergo rapid division and phenotypic alteration during regeneration of the adult liver after injury. Furthermore, hepatocyte-derived proliferative ducts retain the capacity of differentiating back into hepatocytes upon cessation of injury^9^. These observations are supported mostly by the lineage tracing method in genetic mouse models^6,8,10^. The molecular drivers of hepatocyte plasticity that results in clonogenicity and oncogenicity during chronic injury in the adult liver remain elusive.

We previously reported that chronic HCV infection, one of the most potent risk factors for HCC development, induces dedifferentiated phenotypes in liver cells with enhanced expression of cellular stemness-related proteins, such as DCLK1, CD133, AFP, CK19, Lgr5, Lin28, and c-Myc^11^. Among these tumor-related proteins, DCLK1^11–13^ is extensively expressed in the livers of patients with cirrhosis and HCC^11,14–16^. Of note, DCLK1 mRNA and protein is nearly undetectable in normal human or mouse livers^11,17,18^. DCLK1 can also be experimentally induced during liver injury in the DEN/CCl_4_ mouse model of HCC^19^ and in primary human hepatocytes cultured under clonogenic conditions e.g. matrigel. However, DCLK1 expression is not detected in normal human or mouse livers^11,17,18^. Downregulation of DCLK1 inhibits HCV replication, hepatoma cell migration, tumor growth in an HCC xenograft model, EMT, and expression of myeloid-derived S100A9 protein, which suppresses anti-tumor immunity^11,16–18,20–24^. DCLK1 polymerizes tubulins into microtubules and is involved in cargo transport in neuronal cells^13,25,26^. Thus, DCLK1 plays important roles in the regulation of cellular phenotypes.

β-catenin is a critical component of signaling pathways regulating cellular stemness, epithelial polarity and adherens junctions, cytoskeleton remodeling, and tumorigenesis. Aberrant activation of Wnt/β-catenin signaling has been reported in many cancers including HCC (reviewed in Ref.^27^). Typically, β-catenin is ubiquitinated and subjected to proteasomal degradation by a protein scaffold of Axin, APC, GSK-3*β* and CK1, and the E3-ubiquitin ligase b-TrCP in the absence of Wnt signaling. During this process, β-catenin is phosphorylated first at Ser^45^ by CK1, followed by phosphorylation at Ser^33^, Ser^37^, and Thr^41^ by GSK3*β.* However, Wnt binding to its cell surface receptor frizzled (FZ) and co-receptor LRP5/6 inactivates the β-catenin degradation complex. The active, hypophosphorylated β-catenin translocates into the nucleus where it acts as a co-factor for the TCF/LEF family of transcription factors and activates genes involved in cell proliferation, survival, stemness, invasion, and cell cycle regulation. β-catenin also forms a bridge between the cytoplasmic domain of E-cadherin and the cytoskeleton, and is a constituent protein of adherens junctions critical to the establishment and maintenance of epithelial polarity^27^. The microtubule-associated protein PRC1 regulates Wnt signaling by promoting cytoskeletal sequestration of the destruction complex, which results in increased stabilization of cytoplasmic β-catenin^28^.

Because DCLK1 associates with tubulins and regulates microtubule dynamics in addition to being a tumor stem cell protein, we investigated whether DCLK1 promotes hepatocyte plasticity via β-catenin regulation. Here, we report that DCLK1-expressing liver cells show clonogenicity and generate a 48-kDa active β-catenin with preserved unphosphorylated N-terminus due to downregulation of GSK3β activity. This small β-catenin form accumulates in the perinuclear and nuclear regions, associates with transcription factors TCF-4, and activates downstream target cyclin D1. DCLK1-led activation of the atypical β-catenin signaling was also validated in a humanized liver mouse model and liver tissues of patients with cirrhosis and HCC.

## Results

### DCLK1 induces spheroid growth of primary human hepatocytes in 3D suspension culture

**Figure 1:**
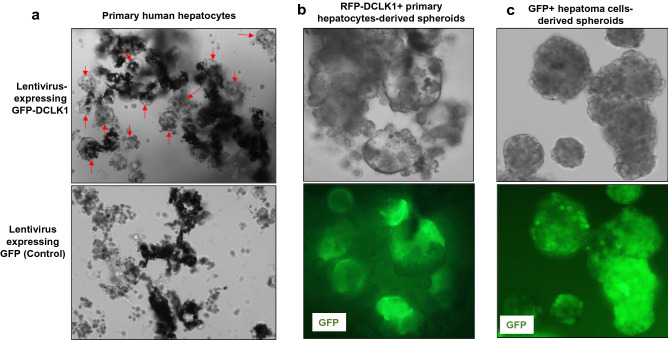
DCLK1-expressing primary human hepatocytes form spheroids in 3D levitated culture devoid of matrigel. (**a**) Monolayer cultures of primary human hepatocytes in complete hepatocyte media were infected with lentiviruses expressing GFP (control) or GFP-tagged human DCLK1 for 48 h. Ten thousand hepatocytes from each trypsinized culture were used for magnetic levitation assay in 6-well ultra-low attachment plates. On day 5, live cell imaging was carried out to record spheroids formation (red arrows, magnification × 10, upper panel). The levitated culture of hepatocytes transduced with lentiviruses expressing RFP (control) is shown in lower panel. (**b**) Live cell images of the spheroids (magnification × 40) in bright field (upper panel)) and for GFP expression (green, lower panel) to show that the spheroids were developed from GFP-DCLK1-expressing primary human hepatocytes. (**c**) GS5 cells derived from Huh7.5 hepatoma cell line and harbor an HCV subgenomic replicon expressing GFP-NS5A were used as a positive control under similar levitation assay conditions.

We previously demonstrated that normal human liver parenchyma stains negatively for DCLK1. However, when primary human hepatocytes from normal livers are cultured in Matrigel, which contains several growth factors and extracellular matrix, some cells form spheroids containing numerous DCLK1 + cells^16^. These spheroids upon further growth contain hepatic cell lineages, such as AFP^+^ hepatoblasts, progenitor/stem-like cells marked by AFP/CK19 co-staining, and albumin-expressing mature hepatocytes. In the present study, we tested whether DCLK1 overexpression induces anchorage-independent spheroid-forming ability in the untransformed primary human hepatocytes in the absence of matrix**.** Hepatocytes derived from normal human liver were cultured on collagen-1-coated plates and infected with lentiviruses expressing GFP (Lenti-GFP) or GFP-tagged human DCLK1 (Lenti-GFP-DCLK1). FACS-based analysis suggested that 12–15% of hepatocytes were transduced after the lentiviral infections and expressed the GFP marker within 48 h (not shown). Similar transduced and subsequently trypsinized cultures formed spheroids in a magnetic levitation assay in which newly formed spheroids grow in suspension culture^29^. As shown in Fig. 1a (upper panel), Lenti-GFP-DCLK1 hepatocytes formed anchorage-independent spheroids growth within one week (highlighted in Fig. 1b). A similar culture of hepatoma cells harboring a GFP tagged HCV NS5A-expressing replicon^11^ that also overexpress DCLK1 was used as a positive control (Fig. 1c). Under similar conditions, Lenti-GFP-infected hepatocytes showed aggregation but without spheroid development (Fig. 1a, lower panel). These observations suggest that DCLK1 overexpression induces anchorage-independent spheroid growth in untransformed primary human hepatocytes.

### DCLK1 overexpression generates a short form of active β-catenin, which increases cyclin D1 expression

β-catenin is an important regulator of clonogenic properties, epithelial cell polarity, and stemness of cells^30^. Therefore, we investigated β-catenin signaling to better understand the role of DCLK1-induced clonogenic properties in liver cells. Hepatoma (Huh7) cells were infected with lentiviruses expressing either RFP or N-terminus RFP-tagged human DCLK1 as described previously^16^. We previously demonstrated that the expressed RFP-DCLK1 protein extensively colocalizes with microtubules, stimulates inflammatory signals, and promotes Huh7 cell migration^16,19^. In this study, live cell imaging revealed the dynamic distribution of RFP-DCLK1 with GFP-labeled microtubules and centrosomes during cell division (Supplementary Fig. S1a) as expected for active DCLK1^26^.

**Figure 2:**
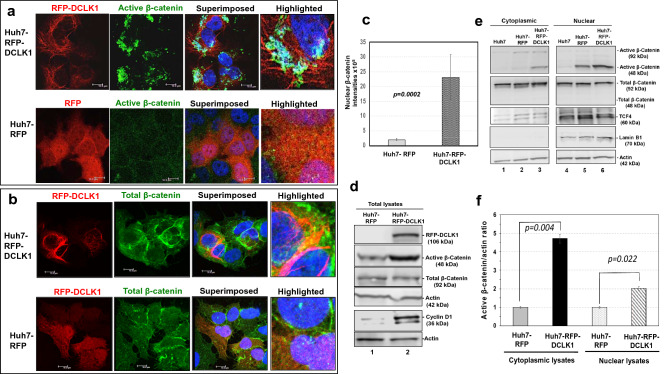
DCLK1 overexpression results in accumulation of 48-kDa hypophosphorylated active β-catenin in hepatoma cells. (**a**) Huh7-expressing RFP-DCLK1 cells (Huh7-RFP-DCLK1) or Huh7-expressing RFP (Huh7-RFP) were stained with anti-active β-catenin (anti-ABC 8E7 mAb, Millipore) and subjected to confocal microscopy. Blue, nuclear stain with Dapi. Scale bar, 10 μm. (**b**) Huh7-RFP-DCLK1 and Huh7-RFP cells were similarly immunostained with antibody (AB19022) against total anti-β-catenin that binds C-terminus and detects phosphorylated and unphosphorylated form of β-catenin. (**c**) Quantitation of nuclear β-catenin in the Huh7-RFP (gray bar) and Huh7-RFP-DCLK1 (hatched bar) using Leica software. The staining intensities for anti-active β-catenin in the nucleus were plotted for both cell-types. *p* value = 0.0002. (**d**) A representative Western blot analysis of total lysates (30 μg each) prepared from Huh7-RFP (control, lane 1) and Huh7-RFP-DCLK1 (lane 2) for expression of active and total β-catenin, and its downstream target protein, cyclin D1. Actin, loading control. (**e**) Detection of cytoplasmic (lanes 1–3) and nuclear (lanes 4–6) levels of active and total β-catenin in parental Huh7 (lanes 1, 4), Huh7-RFP (control, lanes 2, 5), and Huh7-RFP-DCLK1 (lanes 3, 6) cells using Western blot. The blots were probed with antibodies against active and total β-catenin as described above. Mouse mAb (sc-166699) was used for detection of a nuclear protein, TCF-4 that shows enrichment in the nuclear fractions of these cells. Actin and lamin B1, loading controls. The experiment was repeated once to confirmed the results. (**f**) Active β-catenin (48 kDa) bands in cytoplasmic and nuclear fractions of Huh7-RFP (control) and Huh7-RFP-DCLK1 were quantitated using Gel-Quant. The ratios of active β-catenin to actin were adjusted as 1.0 for the Huh7-RFP lysates and compared with band ratios for Huh7-RFP-DCLK1.

Using lentiviruses, we generated Huh7 cells expressing RFP (Huh7-RFP) and RFP-DCLK1 (Huh7-RFP-DCLK1). The cells were immunostained with anti-active β-catenin [ABC(8E7)]. This monoclonal antibody is highly specific and recognizes the N-terminus unphosphorylated HSGATTTAP (aa 36–44) motif of the protein. The β-catenin form detected by this antibody has been shown to be transcriptionally active^31^. Confocal microscopy revealed that active β-catenin was mostly stained in the cytoplasm and nuclear regions of RFP-DCLK1^+^ cells (Fig. 2a, upper panel). However, similar staining pattern for active β-catenin in the nucleus or cytoplasm was not observed for RFP + (lower panel) or Huh7 (not shown) cells, suggesting that the ABC(8E7) staining for active β-catenin was specific for RFP-DCLK1 cells. Growing colonies of Huh7-RFP-DCLK1 cells also showed this staining pattern for active β-catenin (Supplementary Fig. S1b). Quantitation of nuclear staining intensities revealed considerably higher presence of active β-catenin in Huh7-RFP-DCLK1^+^ cells than in Huh7-RFP cells (Fig. 2c). Parallel confocal microscopy was performed for total β-catenin expression in these cells using PLA0230 antibody. This antibody binds to the C-terminus distal region (aa 760–781) of β-catenin and detects full-length (92 kDa) in active and inactive forms. Total β-catenin localization was observed mostly in the cell membrane regions in both cell types (Fig. 2b).

To confirm β-catenin status in these cells, we performed Western blot analysis of total lysates derived from Huh7-RFP and Huh7-RFP-DCLK1 cells (Fig. 2d). While full-length total β-catenin (92 kDa) levels were similar in the lysates, a 48-kDa active β-catenin form was significantly enhanced (2.5 ± 0.5 fold increase) in Huh7-RFP-DCLK1 cells (lane 2) compared with the Huh7-RFP control (lane 1). This increase was accompanied by an enhanced level of cyclin D1 (lane 2), a direct target of active β-catenin. These data suggest that DCLK1 activation may promote cyclin D1-mediated effects in HCC.

Cell fractionation analysis showed a significant increase in 48-kDa active β-catenin in the cytoplasmic and nuclear fractions of Huh7-RFP-DCLK1 cell lysates (Fig. 2e, lanes 3, 6) compared with control (Huh7-RFP, lanes 2, 5) and parent Huh7 lysates (lanes 1, 4). Quantitative analysis of the band intensities revealed a 4.7-fold and twofold increase of 48-kDa active β-catenin in the cytoplasmic and nuclear fractions of Huh7-RFP-DCLK1 cells respectively compared with the corresponding Huh7-RFP cell lysates (Fig. 2f). This 48-kDa band was not detected by either anti-total β-catenin antibodies (PLA0230) (Fig. 2e, second panel from top) or anti-β-catenin (phosphor Y654) antibody (ab59430) (not shown) in the cytoplasmic and nuclear lysates. Of note, the ABC(8E7) did not detect 52–56 kDa β-catenin^32^ in the lysates of these cells (Fig. 2e). These data suggest that the observed 48-kDa active β-catenin represents a major portion of unphosphorylated or hypophosphorylated N-terminus, but lacks the C-terminus regulatory region of the full-length active β-catenin. Nuclear fractions of these cells showed enriched TCF-4 transcription factors compared with the cytoplasmic fractions in all the cell types (Fig. 2e, lower panel). The lamin B1 band intensities for all three cell-types in the nuclear fractions were similar but it was absent in the cytoplasmic fractions (Fig. 2e, fourth panel from top). These results confirmed that the cell fractionation and loading were appropriate.

### DCLK1-overexpressing hepatoma cells exhibit clonogenicity and produce dedifferentiated lineages

**Figure 3:**
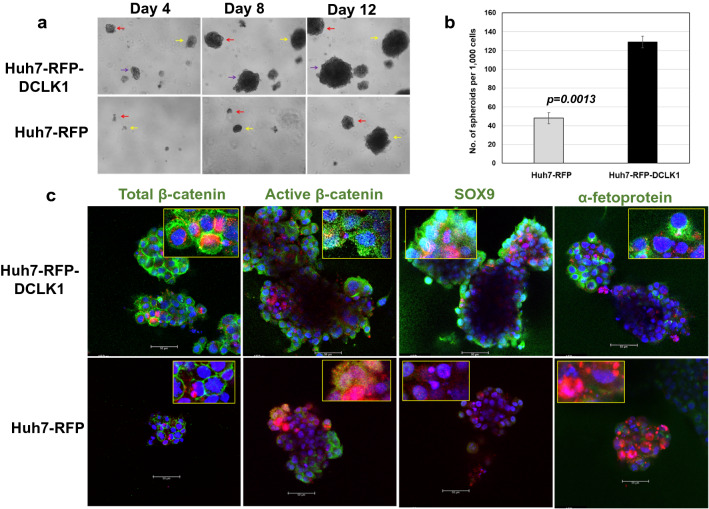
DCLK1-overexpressing hepatoma cells exhibit clonogenicity and produce dedifferentiated lineages. (**a**) Live cell imaging of spheroids derived from Huh7-RFP-DCLK1 (upper panel) and Huh7-RFP cells (lower panel) at × 4 magnification. Single cell suspension of these cells from monolayer cultures were added onto the matrigel layer in Lab-Tek-II 8-well chambered cover slides. The cells were cultured in Hepato-STIM hepatocyte defined media containing EGF. The photographs of developing spheroids (same areas, marked by arrows) were taken at day 4, 8 and 12 post-plating under bright field. (**b**) Quantitation of the number of spheroids formed by 1,000 Huh7-RFP-DCLK1 (black bar) or Huh7-RFP (gray bar) cells. The spheroids were manually counted for each culture. (**c**) Confocal microscopy of spheroids directly immunostained in the matrigel culture. The spheroids derived from Huh7-RFP-DCLK1 (upper panel) or Huh7-RFP (lower panel) were fixed in the matrigel culture and stained for active and total β-catenin, α-fetoprotein, and SOX9 (all shown in green). RFP-DCLK1 or RFP (red) was directly visualized without staining. Blue, nuclear stain with Dapi. Insets, highlighted for positive staining of each protein marker (green).

Huh7-RFP-DCLK1 and Huh7-RFP cells were prepared as single cell suspensions and added to the wells of chambered glass covers containing matrigel to determine clonogenicity of DCLK1-overexpressing cells and cell phenotypes of spheroids. Live images of spheroids derived from these cells were recorded at 4, 8, and 12 days after plating. The results revealed that RFP-DCLK1 overexpression in Huh7 hepatoma cells led to an increase in both the number (2.5 fold) and size of the spheroids (Fig. 3a and b) compared with the Huh7-RFP-derived spheroids. (Fig. 3a, lower panel). Immunofluorescence staining of spheroids directly in the matrigel culture using confocal microscopy showed cells expressing RFP-DCLK1, active β-catenin, α-fetoprotein, and SOX9 (Fig. 3c, upper panel). Total β-catenin staining was mostly localized to the cell membrane (left panel) and was similar to that usually observed in epithelial cells. The results suggest that Huh7-RFP-DCLK1 cells possess clonogenic properties and are capable of producing α-fetoprotein + bipotent progenitor and SOX9 + dedifferented/tumor stem-like cells. The Huh7-RFP cells (control) also formed spheroids, but at lower growth rate, and reduced number of spheroids than Huh7-RFP-DCLK1 (Fig. 3a lower panel). In addition, these spheroids also produced cell lineages with much lower levels of active β-catenin, α-fetoprotein, and SOX9 (Fig. 3c, lower panel), indicating differentiated cell phenotypes.

Taken together, these data (Figs. 2 and 3) suggest that DCLK1-overexpressing cells efficiently generate a 48-kDa form of active β-catenin with preserved unphosphorylated N-terminus, which results in clonogenic and dedifferentiated cellular phenotypes.

### DCLK1 enhances β-catenin signaling via increased phosphorylation of GSK3β-Ser^9^

**Figure 4:**
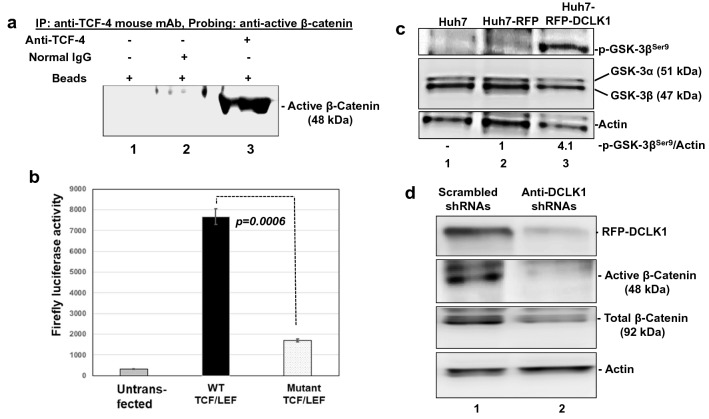
DCLK1 overexpression enhances activation of 48-kDa β-catenin via GSK-3β phosphorylation at Ser^9^. (**a**) Immunoprecipitation of Huh7-RFP-DCLK1 nuclear lysates was carried out with anti-TCF-4 mouse monoclonal (sc-166699) and the blot was probed with anti-ABC 8E7 mAb for active β-catenin. Control immunoprecipitations were carried out with beads alone ((lane 1) and beads plus normal IgG (lane 2) to check specificity of the interaction. (**b**) β-catenin activity assay using TOP/FOP reporter system (Promega) reflects transcriptional activation of β-catenin/TCF-4-dependent luciferase expression in Huh7-RFP-DCLK1 cells. The cells were co-transfected with plasmids expressing firefly luciferase under the control of wild type (WT) or mutant TCF/LEF binding sites in a promoter and pIS2 plasmid that expresses renilla luciferase (transfection control). Untransfected cells were used as negative control. Luciferase was assayed in 10 μg cell lysates (average of three transfection experiments). (**c**) Western blot of total lysates prepared from all three cell-types (as indicated) using a mouse monoclonal antibody (mAb) sc373800 (Santa Cruz) that detects phospho-GSK-3β^Ser9^ (upper panel). Total GSK-3α and GSK-3β were detected by sc-7291 monoclonal antibody (Santa Cruz). Relative phospho-GSK-3β^Ser9^ to actin band intensities are shown at the bottom. These results were confirmed by a similar Western blot analysis. (**d**) shRNA-mediated downregulation of DCLK1 reduces active 48-kDa β-catenin form. Huh7-RFP-DCLK1 cells were infected with lentiviruses encoding our previously validated anti-DCLK1 shRNA (lane 2) or scrambled shRNA (lane 1)^16^. The lysates (30 μg each) were subjected to Western blots for DCLK1, total β-catenin, and active β-catenin.

Since nuclear accumulation of the 48-kDa form of active β*-*catenin was observed in DCLK1 + cells, we determined its interaction with the transcription factor TCF-4 by immunoprecipitation (IP) using nuclear lysates of Huh7-RFP-DCLK1 cells. IP with anti-TCF-4 antibody followed by probing blots with anti-ABC(8E7) showed binding of the 48-kDa form of active β*-*catenin with nuclear TCF-4 (Fig. 4a, lane 3), suggesting that the short form retains a TCF-binding site within the armadillo (Arm) repeats (Arm 4 through 8). We next employed a luciferase-based reporter assay to determine whether the 48-kDa β*-*catenin-TCF-4 interaction activates a downstream target gene. The β-catenin activity in Huh7-RFP-DCLK1 cells were measured by transient transfection of TOPFlash (wild-type promoter-Luc) or FOPFlash (mutant promoter-Luc) luciferase (Luc) reporter plasmids. The results showed that TCF-4/LEF-responsive wild type promoter activities were significantly enhanced (fourfold) compared with the mutant TCF/LEF promoter in Huh7-RFP-DCLK1 cells (Fig. 4b). These observations clearly suggest that the interaction of the small 48-kDa form of β*-*catenin is capable of activating TCF-4-dependent transcription of target genes.

In the absence of Wnt signaling, GSK-3β phosphorylates the N-terminus of β*-*catenin at Ser^33^, Ser^37^, and Thr^45^ residues for proteasomal degradation^33^. However, GSK-3β kinase activity is inhibited due to Ser^9^ phosphorylation by several kinases (e.g., AKT, p70S6, p90Rsk), leading to stabilization of β-catenin^34^. To understand this dynamic in our cell lines, we next performed Western blot analysis of total cell lysates derived from the three cell-types described above. Huh7-RFP-DCLK1 cells demonstrated an increased Ser^9^ phosphorylation of GSK-3β (Fig. 4c, lane 3, top panel) as compared to the corresponding controls (lanes 1, 2), suggesting that overexpression of DCLK1 may inhibit classic proteasome degradation of β-catenin due to downregulation of GSK-3β kinase activity. Total GSK-3α and GSK-3β levels were not affected by RFP or RFP-DCLK1 expression in these cells (middle panel). To confirm whether a DCLK1-dependent mechanism generates the 48-kDa active β-catenin, we repeated this analysis using a validated anti-DCLK1 shRNA as an inhibitor^16^. Downregulation of DCLK1 resulted in a dramatic reduction of the 48-kDa form of active *β*-catenin form (Fig. 4d, lane 2).

Taken together, these experiments show that DCLK1 activates β-catenin (48-kDa) signaling. In addition, GSK-3β activities that are required for N-terminus hyperphosphorylation-mediated degradation of β-catenin are downregulated due to increased GSK-3β-Ser^9^ phosphorylation in DCLK1-overexpressing hepatoma cells.

### Bortezomib increases 48-kDa active β-catenin but not the full-length protein in DCLK1-overexpressing cells

**Figure 5:**
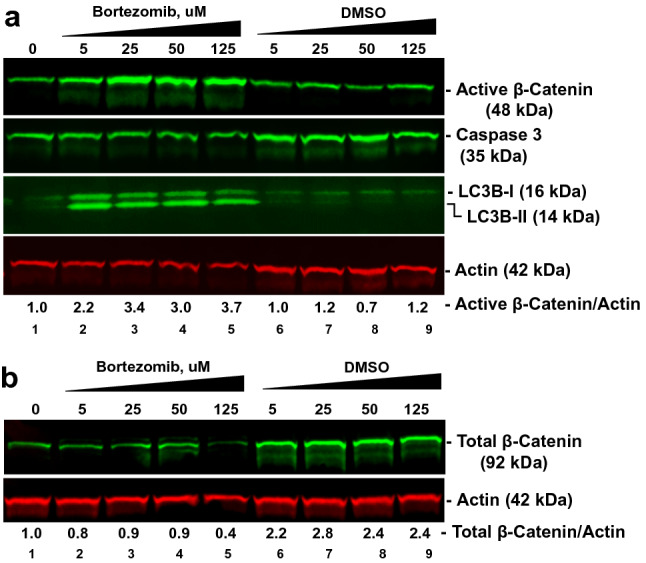
DCLK1 induces 48-kDa active β-catenin in hepatoma cells by proteasome-independent mechanism. (**a**) Huh7-RFP-DCLK1 cell cultures were treated with increasing amounts (5 µM to 125 µM, lanes 2–5) of bortezomib (proteasome inhibitor), or equivalent amounts of its solvent (DMSO, lanes 6–9). Lane 1, Untreated control. Total lysates were subjected to Western blot analysis for active β-catenin, apoptosis marker (caspase 3 cleavage) and autophagy marker (LC3B cleavage). (**b**) Expression levels of total β-catenin in bortezomib treated and untreated Huh7-RFP-DCLK1 cells. The ratios of band intensities of active or total β-catenin to actin (loading control) were calculated using ImageStudio software and relative values are shown at the bottom of the figures.

β-catenin is a target of proteasomal processing and degradation. Bortezomib inhibits proteasomal activities, and triggers cell-death by inducing apoptosis and autophagy pathways^35^. To determine whether the 48-kDa form of active β-catenin is generated by proteasomal activities, Huh7-RFP-DCLK1 cells were treated with increasing amounts (5 µM to 125 µM) of bortezomib or equivalent amounts of solvent (DMSO) for 48 h. Bortezomib treatment of Huh7-RFP-DCLK1 cells caused increased accumulation of 48-kDa active β-catenin (Fig. 5a, lanes 2–5) compared with untreated control (lane 1). This effect was not observed for DMSO controls (lanes 6–9). The biological effects of bortezomib on these cells was confirmed by the activation of autophagy, as indicated by cleavage of LC3B-I (third panel from top, lanes 2–5) compared with the untreated (lane 1) and DMSO-treated cells (lanes 6–9). Full-length total β-catenin (92 kDa)/actin ratios were similar in the bortezomib-treated and untreated cells (Fig. 5b, lanes 2–4), suggesting that proteasome inhibition did not affect levels of full-length β-catenin. At all the DMSO treatment concentrations, total β-catenin (92 kDa) levels were similar (lanes 6–9). Bortezomib is also known to induce caspase 3 cleavage, which is indicative of apoptosis. However, the levels of full-length caspase 3 were similar in the bortezomib-treated and untreated Huh7-RFP-DCLK1 cells. These results suggest that the level of 48-kDa active β-catenin is regulated by a proteasome-independent mechanism.

### DCLK1-positive cells express 48-kDa active β-catenin in livers of patients with cirrhosis and HCC

**Figure 6:**
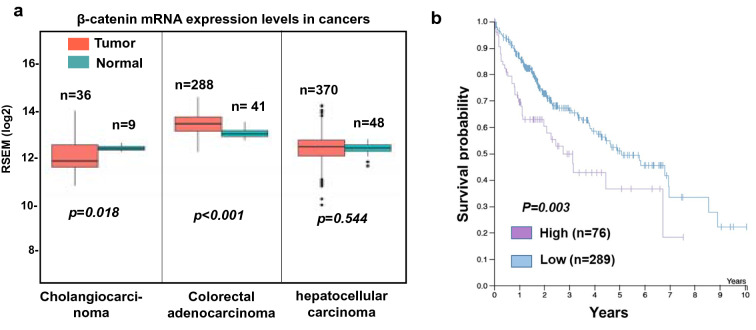
β-catenin mRNA expression increases in colorectal adenocarcinoma but not in liver cancers (HCC and cholangiocarcinoma). (**a**) TCGA database for β-catenin mRNA expression levels in HCC (n = 370, p = 0.544), cholangiocarcinoma (n = 36, p = 0.018) and colorectal adenocarcinoma (n = 288, p < 0.001) were analyzed and the mRNA levels for each cancer were expressed as RSEM (log2) values. The data were normalized with respective normal tissues. (**b**) Survival plot was generated for high and low β-catenin mRNA-expressing HCC patients (*p* = 0.003) using the TCGA data.

A large percentage (60%-70%) of patients with cirrhosis and HCC express DCLK1 in their livers and this expression correlates with a worse prognosis^14,16^. To assess whether DCLK1-mediated alteration in β-catenin correlated with clinical activity, we first analyzed the TCGA database for β-catenin mRNA expression levels in HCC, cholangiocarcinoma, and colorectal adenocarcinoma. The expression levels were normalized using normal tissues. There was increased β-catenin mRNA in colorectal cancers (*n* = 288, *p* < 0.001) compared with normal samples (*n* = 41, Fig. 6a). However, β-catenin mRNA levels in HCC (*n* = 370, *p* = 0.544) and cholangiocarcinoma (*n* = 36, *p* = 0.018) showed variable expression compared with levels found in normal tissues. The survival patients with HCC was significantly reduced with high expression (n = 76, Fig. 6b), suggesting that levels of β-catenin is likely to influence clinical outcomes.

**Figure 7:**
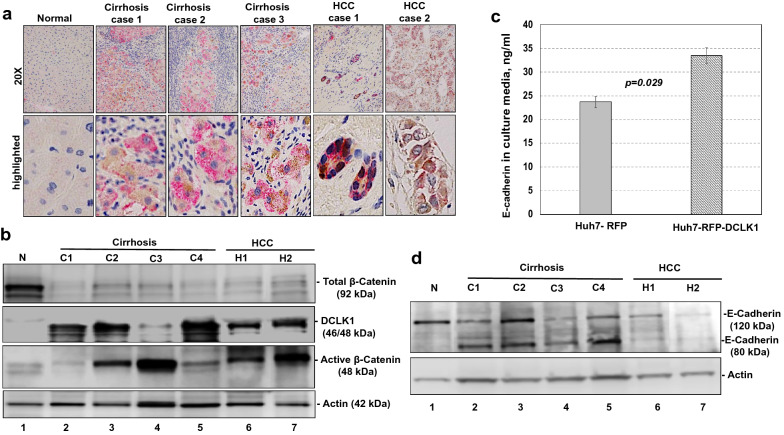
Livers of HCV and HBV patients with cirrhosis and HCC exhibit activation of DCLK1-β-catenin signaling with increased migratory cell phenotypes. (**a**) Immunohistochemical staining of liver tissues (normal, HCV + cirrhosis, and HCV + HCC) was simultaneously carried out with anti-ABC 8E7 mAb and anti-DCLK1 ab109029 antibodies. The co-staining of DCLK1 (brown) and active β-catenin (red) in the same epithelial cells are highlighted in the lower panel. Similar staining for HBV-positive cirrhosis and HCC are shown in Supplementary Fig. S2. (**b**) Total lysates (40 μg) of liver tissues from HCV-positive patients (n = 6) with cirrhosis (lanes 2–5) and HCC (lanes 6, 7), and normal liver (lane 1) were subjected for Western blot using antibodies against active and total β-catenin, DCLK1 (ab109029), and actin. (**c**) Quantitation of soluble E-cadherin level in the media supernatants of Huh7-RFP (gray bar) and Huh7-RFP-DCLK1 (hatched bar) using ELISA kit (Thermo Fisher) after 24 h of cell passage. (**d**) Western blot analysis of the total lysates of liver tissues as described in section (**b**) using mouse mAb sc-8426 that detects both full-length (120-kDa) and cleaved/soluble ectodomain (80-kDa) of E-cadherin.

To determine whether active β-catenin and DCLK1 are expressed in the same liver cells, we performed co-immunohistochemical staining of liver tissues from patients with cirrhosis and HCC (n = 20). The immunohistochemistry results revealed specific co-staining of active β-catenin (red) and DCLK1 (brown) in the same epithelial cells (Fig. 7a, five representative cases are shown). Healthy/normal liver did not display staining for DCLK1 or active β-catenin (top left panel) (also see 1 normal and 4 HBV cases in Supplementary Fig. S2). Next, we determined relative expression of DCLK1, and active and total β-catenin in the total lysates prepared from liver tissues of patients with cirrhosis and HCC (Fig. 7b). We observed a clear, upregulated expression of DCLK1 and 48-kDa active β-catenin in these patients (lanes 3–7). Although C3 lysate (lane 4) showed weak DCLK1 band, staining of a similar blot with an another anti-DCLK1 antibody (ab31704) revealed 48-kDa and 45 kDa DCLK1 bands, most likely representing alternatively spliced isoforms (not shown). Patients’ liver tissues exhibited downregulation of full-length total β-catenin (92 kDa) compared with normal liver (lane 1). Interestingly, patient C1 (lane 2) did not exhibit any correlation between DCLK1 and active β-catenin, suggesting that DCLK1 expression may have influenced other tumorigenic stimuli in this case.

Cleavage of full-length E-cadherin (120-kDa) by a number of proteinases at the cell surface produces soluble 80-kDa ectodomain, which reduces epithelial features and induces migratory phenotypes due to disruption of cell–cell interactions^36–38^. We determined soluble E-cadherin levels in the cell culture supernatant of DCLK1-overexpressing hepatoma cells to determine whether DCLK1 overexpression in the liver diseases is related to change in epithelial phenotypes of the cells. Within 24 h of cell passage, we observed a modest increase in the cleaved/soluble E-cadherin level in the media supernatant of Huh7-RFP-DCLK1 cells compared with the media of Huh7-RFP cultures (Fig. 7c). Western blot analysis revealed two predominant forms (120-kDa and 80-kDa) of E-cadherin in cirrhotic and HCC liver lysates (Fig. 7d, lanes 2–7). This 80-kDa E-cadherin form was absent from normal liver tissues (lane 1). These results show that hepatic epithelial cells acquire DCLK1 + /active β-catenin + phenotypes in cirrhosis and HCC, and is accompanied by appearance of cleaved E-cadherin. In H1 and H2 cases (Lanes 6, 7), the expression of both forms of E-cadherin was milder than cirrhosis because of extensive loss of epithelial cells as determined by H&E staining (not shown). These changes would suggest a loss of the epithelial polarity, acquisition of migratory phenotypes, and enhanced neoplastic characteristics of the cells after DCLK1 overexpression.

### DCLK1 mediates activation of β-catenin in humanized FRG mouse livers

**Figure 8:**
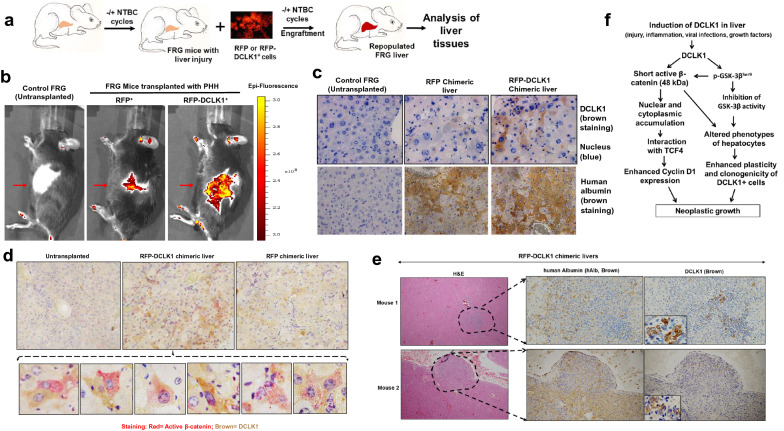
DCLK1-expressing primary human hepatocytes shows activation of β-catenin in humanized Fah^−/−^/Rag2^−/−^/Il2rg^−/−^ (FRG) mouse livers. (**a**) Scheme of NTBC cycle and hepatocyte transplantation into the FRG mice. (**b**) RFP or RFP-DCLK1-expressing primary human hepatocytes (1 million each) transplanted into the spleen of FRG mice after removing hair (red arrow, clean area). The mice were imaged live for RFP expression 10 days post-transplantation with IVIS Spectrum Imager. Control, FRG mice at ± NTBC water cycles but without transplantation (left panel); images of a representative transplanted FRG mouse are shown in each group. Red to yellow scale indicates increase in epi-fluorescence intensities. (**c**) Immunohistochemistry of human-mouse chimeric FRG livers were carried out for human albumin (hAlb, ab2406) (lower panel, brown) and DCLK1 (ab109029) (upper panel, brown) 6 weeks post-transplantation. Control, FRG mice treated at ± NTBC water cycles similar to other mice but without transplantation of hepatocytes (left panel). Blue, nuclear stain. (**d**) Co-staining of active β-catenin (red) and DCLK1 (brown) in the chimeric (RFP and RFP-DCLK1) and control (untransplanted) FRG livers. Lower panel, hepatocytes highlighted for nuclear and cytoplasmic expressions of active β-catenin (red) in DCLK1 + cells. The controls (RFP-transplanted and untransplanted FRG livers) lack this co-expression pattern. (**e**) Distinct proliferation of RFP-DCLK1 + human hepatocytes in the humanized FRG livers confirmed by DCLK1 and hAlb expression (left pane). H&E staining, left panel. (**f**) Schematic presentation of DCLK1 signaling-mediated activation of the short β-catenin (48-kDa), which contributes to hepatocytes plasticity and neoplastic growth.

We next investigated DCLK1 and β-catenin expression in vivo using an animal model that recapitulates liver injury and propagation of human hepatocytes. Fumarylacetoacetate hydrolase (FAH) deficiency in the immunodeficient FRG mouse results in liver damage due to accumulation of toxic tyrosine catabolites. However, NTBC (nitisinone) prevents liver damage in these mice by blocking 4-hydroxyphenylpyruvate (4-HPD) two steps upstream of FAH in the tyrosine catabolic pathway^39^. Using this model, we cyclically withdrew NTBC from their drinking water and intrasplenically transplanted primary human hepatocytes anticipating that these cells would expand and repopulate mouse livers with the human hepatocytes (scheme in Fig. 8a). This process created a chimeric mouse model in which to investigate the role of DCLK1 expression in vivo.

RFP or RFP-DCLK1 expressing primary human hepatocytes were transplanted into FRG mice (5 mice per group). The RFP fluorescence was clearly detected mostly in the abdominal region 10 days post-transplantation for both RFP and RFP-DCLK1 groups (Fig. 8b, indicated with red arrow). After euthanasia, the mouse livers were subjected to immunohistochemistry for human albumin (hAlb), DCLK1, and active β-catenin. RFP-DCLK1 chimeric mice showed extensive staining for hAlb and DCLK1 (Fig. 8c, right panel, only one representative section is shown). The RFP chimeric liver exhibited hAlb but rare DCLK1 + cells with weak staining (middle panel). In RFP-DCLK1 chimeric liver, we also noticed proliferative nodules staining positive for DCLK1 and hAlb (Fig. 8e). The control (untransplanted) mouse livers were negative for both DCLK1 and hAlb (Fig. 8c, left panel), indicating that liver injury alone due to FAH deficiency is not sufficient to induce DCLK1 in these mice. Together, these results suggest that DCLK1-expressing human hepatocytes can repopulate into the FRG liver microenvironment.

To further determine whether the human hepatocytes exhibit the potential to adopt more immature or dedifferentiated phenotypes, we examined active β-catenin expression in the DCLK1-expressing cells by co-immunostaining. A number of DCLK1 + hepatocytes exhibited cytoplasmic and nuclear accumulation of active β-catenin in RFP-DCLK1 chimeric livers but not in RFP control livers (Fig. 8d, highlighted in lower panel). Staining of the FRG livers for full-length total β-catenin revealed localization mostly in the cell membrane in both RFP-DCLK1 and RFP chimeric mice (Supplementary Fig. S3, left panel). However, active β-catenin staining was only seen in the nucleus and cytoplasm of RFP-DCLK1 chimeric liver (right panel), suggesting that the observed active β-catenin in RFP-DCLK1 chimeric liver lacks C-terminus but retains a hypophosphorylated N-terminus. This finding is reminiscent of the results from the in vitro experiments. Taken together, these results clearly suggest that DCLK1-overexpressing primary human hepatocytes exhibit a propensity to adapt undifferentiated phenotypes due to activation of β-catenin and can propagate in the liver microenvironment.

## Discussion

Hepatocellular carcinoma (HCC) predominantly originates from hepatocytes in murine models^3,40,41^. The precise molecular mechanisms that regulate tumorigenesis and neoplastic conversion of normal hepatocytes after chronic liver injury remain unclear. In this report, we present evidence for a novel DCLK1-regulated cell-signaling pathway that contributes to clonogenicity and oncogenicity of hepatic epithelial cells by activating a 48-kDa form of β-catenin. Under normal physiologic conditions, β-catenin activity is minimal in periportal hepatocytes. However, chronic liver injury activates β-catenin and this stimulates self-duplication and/or transdifferentiation of hepatocytes to generate new cells^10^. β-catenin is a critical component of the Wnt signaling pathway and the cell surface cadherin adherens complex^27^. Upon activation, β-catenin translocates into the nucleus where it acts as a co-factor for TCF/LEF transcription factors and activates downstream target genes such as c-Myc, cyclin D1, survivin, and growth factors which co-ordinate the restoration of normal hepatic architecture and liver physiology^27^. β-catenin also regulates SOX9, a transcription factor that marks tumor stem cells and bipotent progenitors after liver injury and produces both hepatocytes and ductal cells for liver repair ^42^. Here, we demonstrate that DCLK1-overexpressing hepatoma cells develop into spheroids composed of SOX9 + and α-fetoprotein + cells. These results suggest that DCLK1 + hepatoma cells possess clonogenic capacity and produce dedifferentiated hepatic cell lineages likely by activating non-canonical/atypical β-catenin signaling. The DCLK1-expressing hepatoma cells produce a short form of active β-catenin containing an unphosphorylated N-terminus and a preserved TCF-4 binding capacity, but do not have the C-terminus regulatory region of the full-length β-catenin (92 kDa) that is required to bind E-cadherin. This short β-catenin form accumulates in the nucleus and activates TCF-responsive genes, such as cyclin D1. Increased cyclin D1 expression leads to hepatocyte transformation in a transgenic mouse model of HCC^43^. It has also been reported to correlate with an aggressive form of HCC in patients^44^. The Huh7 hepatoma cells used in this study have been shown to have little or no expression of *Wnt* family members. The tumor-promoting mutations in *CTNNB1 (*β-catenin), *APC*, or *AXIN* genes are also absent in this cell line. Interestingly, β-catenin activation is still required for optimal growth of Huh7 cells. However, the mechanism that regulates this activation is unknown^45^. Our results provide a novel non-canonical/atypical mechanism for activation of β-catenin by DCLK1 (illustrated in Fig. 8f).

Protease digestion of full-length β-catenin with trypsin and subtilisin generates 40-kDa (134–550 aa containing armadillo motifs) and 10-kDa (551–671 aa) fragments^46^. Recently, Goretsky et al.^32^ demonstrated the existence of a low molecular weight (52–56 kDa) β-catenin that was phosphorylated at Ser^552^ in colon cancers. This form, generated by a proteasome-dependent mechanism, lacks the significant parts of both N- and C-termini but retains armadillo repeats that interacts with TCF4. In DCLK1-overexpressing hepatoma cells and livers derived from patients with cirrhosis and HCC, we detected a distinct low molecular weight (48-kDa) β-catenin with an intact N-terminus that was not phosphorylated at Ser^33^ and Ser^37^. This form was repeatedly detected in the lysates of cultured cells and liver tissues containing protease/phosphatase inhibitor cocktails (Sigma and Abcam). Phosphorylation of Ser^33^ and Ser^37^ residues by GSK-3β is a critical early event during proteasomal degradation of β*-*catenin. Overexpression of DCLK1 in Huh7 cells leads to phosphorylation of GSK-3β at the Ser^9^ residue (Fig. 4c), which inhibits its kinase activity. We predict that reduced GSK-3β activity due to increased Ser^9^ phosphorylation in DCLK1-overexpressing cells will result in accumulation of β-catenin with an intact N-terminus. In order to investigate this further, we treated DCLK1-overexpressing Huh7 cells with the proteasome inhibitor (bortezomib). Indeed, we observed accumulation of 48-kDa β-catenin and no change in total full-length β-catenin in the bortezomib-treated Huh7-RFP-DCLK1 cells (Fig. 5). These data support the notion that this 48-KDa form was not generated by proteasome processing. Although the precise mechanism of generation of this β-catenin form is under investigation, we nevertheless observed its interaction with TCF4 and the downstream activation of the target genes. Database analysis revealed variation (p = 0.544) in the β-catenin mRNA levels in the livers of HCC patients (n = 370) compared to the normal livers (Fig. 6a). Higher expression of β-catenin mRNA levels in patients (n = 76) correlated worse prognosis for the HCC patients (Fig. 6b). It is possible that post-transcriptional regulations, which are not reflected by the mRNA levels alone, also contribute to the HCC outcomes. However, this will require additional investigation. Taken together, these data suggest that DCLK1 promotes the generation of a novel 48-kDa β-catenin by inhibition of GSK-3β activities.

In a previous study, we demonstrated that endogenous DCLK1 is induced in primary human hepatocytes-derived spheroids cultured in Matrigel, which is a rich source of tumor-derived murine extracellular matrix and growth factors^47^. Further analysis showed that these spheroids contain AFP/CK19-positive undifferentiated and albumin-positive mature hepatocytes^16^. A similar observation was made for AFP expression in the Huh7-RFP-DCLK1 cells-derived spheroids (Fig. 3c). To evaluate the role of DCLK1 in neoplastic initiation, we overexpressed DCLK1 in primary human hepatocytes. Of note, normal mouse and human hepatocytes do not express DCLK1^11,14–16^. However, there is a marked increase in hepatic DCLK1 expression after liver injury, cirrhosis, and HCC. DCLK1 overexpression in untransformed primary human hepatocytes resulted in spheroid growth in 3D levitated cultures that were devoid of Matrigel, compared with control cells, which formed aggregates but no spheroids (Fig. 1). We also confirmed clonogenic properties of DCLK1 + hepatoma cells and its lineages (Fig. 3). These data suggest that overexpression of DCLK1 results in the acquisition of self-renewal capacity in differentiated hepatocytes and hepatoma cells. This may represent a DCLK1-dependent dedifferentiation or transdifferentiation towards a more immature cell-type, reflecting reprogramming due to DCLK1-mediated hepatocyte plasticity. We utilized an FRG mouse model to further test this observation in vivo. The FRG mouse liver can be extensively repopulated (> 80%) by the robust expansion of transplanted human hepatocytes to produce humanized/chimeric mouse liver^39,48^. This approach allowed us to examine the β-catenin expression in DCLK1-positive human hepatocytes propagating in a liver microenvironment. We observed immunoreactive active β-catenin in the cytoplasm and nuclei of the transplanted human hepatocytes overexpressing DCLK1. Interestingly, the antibody that specifically binds the C-terminus of the full-length β-catenin did not stain for cytoplasmic and nuclear β-catenin (Supplementary Fig. S3, left panel). These results suggest that the active β-catenin observed in the chimeric liver lacks the C-terminus but contains unphosphorylated N-terminus. Immunohistochemical analysis revealed hepatic cells positive for DCLK1 and active β-catenin only in the RFP-DCLK1-humanized mouse livers, but not in the corresponding control. Rarely, we observed adjacent hepatocytes where one cell expressed DCLK1 and the adjacent cell expressed both DCLK1 and active β-catenin (Fig. 8d, lower panel). This finding could represent DCLK1 cells giving rise to daughter cells that expresses active β-catenin or could represent trans-/de-differentiation. Further studies are underway to address the significance of these findings. In addition, HBV or HCV-induced hepatitis and restoration of immune system in the humanized FRG mice are required to broaden the findings presented here.

DCLK1 maintains the cytoskeleton by regulating microtubule dynamics, and thereby plays critical roles in mitosis, cell motility, and intracellular protein transport^25,49^. β-Catenin is an important component of the adherens junctions where it bridges E-cadherin to α-catenin and the actin cytoskeleton to maintain epithelial polarity and intercellular adhesion. Cleavage of the E-cadherin ectodomain near the plasma membrane by α-secretase, matrix metalloproteinases (MMP-2/3/7/9/14) and cathepsins, among others, generates a soluble 80-kDa E-cadherin fragment in the extracellular space, disassembles the cadherin–catenin complex, and releases β-catenin into the cytoplasm (reviewed in Ref.^36^). We previously reported that DCLK1 expression in HCC strongly correlates with MMP2 and MMP14^50^, which are involved in E-cadherin cleavage. Unlike normal livers, which lack both DCLK1 and 80-kDa E-cadherin, livers from patients with cirrhosis and HCC clearly showed both proteins (Fig. 7d). Based on antibody mapping and primary structure analysis, we suspect that the observed 48-kDa form of active β-catenin lacks an E-cadherin binding site. We observed a modest increase in the soluble E-cadherin level of Huh7-RFP-DCLK1 cells as compared with Huh7-RFP-derived media supernatant within 24 h of cell passage (Fig. 7c). It is possible that DCLK1-dependent generation of short active β-catenin and reduction in its full-length form in the patient livers also results in destabilization of adherens junctions that ultimately alters the epithelial polarity.

In conclusion, our data demonstrate a novel role of DCLK1 in the regulation of a non-canonical/atypical β-catenin signaling pathway. This signaling may be responsible for the chronic injury-mediated hepatocyte oncogenicity that precedes neoplasia.

## Methods

### Cell lines, human hepatocytes, human liver tissues, and animal models

The cell lines (Huh7and its derivatives, HepG2) and cryopreserved primary human hepatocytes (obtained from Corning) were tested negative for mycoplasma and viruses using h-IMPACT panel. All methods were carried out in accordance with the guidelines and regulations of the University of Oklahoma Health Sciences Center (OUHSC) Institutional Biosafety Committee (approval #200470-2440A). The normal and diseased human liver tissues were obtained from the Liver Tissue Cell Distribution System (LTCDS, Minnesota). Informed consent of donors (age > 18 years) was obtained by LTCDS. The use of human cells and tissues was approved by OUHSC Institutional Review Board (approval #5710). Fah^−/−^/Rag2^−/−^/Il2rg^−/−^ (FRG) mice with C57BL/6 background were purchased from Yecuris (Oregon). The mouse studies were conducted in compliance with the protocol approved by OUHSC Institutional Animal Care and Use Committee (approval #15-083-HIC-H).

### Expression of DCLK1 in primary human hepatocytes and hepatoma cells

Lentiviruses expressing RFP- or GFP-tagged human DCLK1 (isoform 1) were constructed using Addgene vectors, as described previously^16^. The lentiviruses expressing RFP, GFP, RFP-DCLK1, and DCLK1-GFP were produced and purified as described by Tiscornia et al.^51^. The human hepatocytes were cultured on collagen I-coated plates in Hepato-STIM defined medium supplemented with EGF (10 ng/mL), 2 mM l-glutamine, and 1X antibiotic–antimycotic, and were infected with lentiviruses (~ MOI 20–30). The RFP or GFP expression was monitored in live cells by fluorescence microscopy. The GFP- or RFP-expressing cells were enriched or analyzed using the FACS method. Huh7 cells were cultured in Dulbecco’s Modified Eagle’s Medium (DMEM) supplemented with 1 × Pen/Strep, 1 × non-essential amino acids, and 10% fetal bovine serum (all from Invitrogen), and were maintained at 37 °C and 5% CO_2_. Huh7 cells expressing RFP (Huh7-RFP) and RFP-DCLK1 (Huh7-RFP-DCLK1) were prepared by infection with the lentiviruses, followed by enrichment using the FACS method. Knockdown of DCLK1 in cells using a highly effective shDCLK1-291 shRNA and its scrambled negative control have been previously described^16^. The media supernatants of the cells were collected 24 h post-passage and clarified for cell debris by centrifugation. Soluble E-cadherin levels in the culture supernatants were estimated by Quantikine Human E-cadherin ELISA protocol (R&D Systems). The cell lines (Huh7 and its derivatives) and primary human hepatocytes (obtained from vendors) were tested negative for mycoplasma, lymphotropic choriomeningitis virus and hantavirus using h-IMPACT panel.

### Immunofluorescence, confocal microscopy and fluorescence-activated cell sorting (FACS)

Cells (Huh7, Huh7-RFP, Huh7-RFP-DCLK1) grown on glass cover-slips were rinsed briefly in phosphate-buffered saline (PBS), fixed in 4% paraformaldehyde/PBS pH 7.4 for 20 min at room temperature, washed twice with ice-cold PBS, and permeabilized in ice-cold acetone. Cells were incubated with blocking buffer (10% serum, 0.01% Triton X-100, in PBS, pH 7.4) for 1 h, washed with PBS, and treated with anti-active β-catenin (anti-ABC, clone 8E7, Cat# 05–665) or total anti-β-catenin (Cat# PLA0230, Sigma) antibodies in PBS-T containing 1% BSA for 2 h at room temperature. After thorough washing with PBS-T, cover slips were incubated in the appropriate AlexaFluor-conjugated secondary antibodies. The nuclei were counterstained with DAPI (0.1–1 μg/mL PBS). The cover slips were mounted on microscope slides in ProLong Gold and subjected to confocal microscopy. The RFP- and RFP-DCLK1-expressing cultured cells were subjected to FACS analysis using standard method. Immunohistochemistry was performed according to manufacturer’s protocol using the Leica Bond-III™ Polymer Refine Detection system (DS 9,800). The β-catenin activity assay in RFP-DCLK1^+^ primary hepatocytes were measured by transiently transfected with the TOPFlash or FOPFlash plasmids as described in the Promega’s protocol.

### Lysate preparations and Western blot

The nuclear and cytoplasmic fractions of cultured cells were prepared using NE-PER reagents (Cat #78833, Thermo Fisher). Whole cell lysates were prepared using RIPA buffer (Thermo Fisher). The lysates were prepared using Bullet Blender’s protocol (Next Advance, Inc.). All lysates were treated with protease/phosphatase inhibitor cocktail (Abcam or Sigma). Western blots were carried out with chemiluminescence or fluorescence reagents using LI-COR imaging system. The band intensities were calculated using Image Studio Digits. The images were produced in compliance with the digital image and integrity policies.

### Spheroid assays

Primary human hepatocytes were cultured on collagen I-coated plates in complete Hapato-STIM hepatocyte medium containing EGF (Corning). For levitated spheroid cell culture, the GFP + or RFP + human primary hepatocytes were isolated by FACS. Primary human hepatocytes (~ 20,000, per well) were resuspended in complete Hepato-STIM medium, mixed with lentiviruses, and incubated in a six-well ultra-low attachment plate for 2 h in a CO_2_ incubator. Nanoshuttle beads (n3D Biosciences) were then mixed with suspended cells. The plates were covered with a magnet-cover top, incubated at 5% CO_2_ at 37 °C, and were photographed under phase-contrast microscopy at various time points to monitor spheroid growth in suspension culture. Similar cultures incubated for 48 h were used for determination of transduction efficiency using the FACS method.

One thousand Huh7, Huh7-RFP or Huh7-RFP-DCLK1 cells were cultured in a 24-well low attachment plate containing RPMI glutamax medium plus 1% fetal calf serum in matrigel for one week and total number of spheroids per well were counted manually. For live imaging of the spheroids, the cells were cultured in Lab-Teck II 8-well chambered cover glass system (Thermo Fisher) containing 100 µl matrigel per well in Hepato-STIM hepatocyte defined medium supplemented with EGF(10 ng/mL). The developing spheroids were directly imaged (× 4 magnification) at day 2, 4, 8, and 12 of plating the cells. To perform confocal microscopy, these spheroid cultures were fixed with 10% formamide-PBS for 20 min, blocked with 1% BSA in washing buffer (PBS, 0.5% Triton X-100, 0.05% Tween-20). After washing three times, the spheroids were stained with primary and secondary antibodies. Nuclear staining was carried out with DAPI. The spheroids were subjected to confocal microscopy after washing and addition of one drop of ProLong Gold Anti-fade reagent (Invitrogen) directly into the wells.

### Transplantation of primary human hepatocytes into FRG mice

FRG mice with a C57BL/6 background (Yecuris, Oregon) were randomized into three groups (untransplanted, RFP + cells transplanted, and RFP-DCLK1 + cells transplanted). The mice were kept healthy with 5LJ5 diet and sterile drinking water containing nitisinone (NTBC, 8 mg/L) and 3% dextrose. The mice were subjected to a defined NTBC ON/Off cycle according to Yecuris’s manual, followed by infection with adenovirus expressing urokinase-type plasminogen activator (Ad:uPA, Yecuris Cat# 20-0029) administered by retro-orbital injection (1.25E + 9 pfu in 100 microL of PBS) under anesthesia one day before hepatocyte transplantation. Human hepatocytes (100,000 cells in 200 μL of culture medium) were manually injected into the spleens. Prophylactic treatment with sulfamethoxazole (640 μg/mL) and trimethoprim (128 μg/mL) in drinking water was administered to the FRG mice once every two weeks to reduce the risk of bacterial infection. The mice were imaged for RFP expression 10 days post-transplantation with a IVIS Spectrum Imager. Upon termination (5 weeks post-transplantation), the livers were subjected to immunohistochemical staining.

### Statistical analysis

All experiments were performed in biological replicates of at least three and repeated to confirm the results. The graphs were presented as mean ± standard deviation. The *p*-values were calculated using Student’s *t*-test. Results with *p* ≤ 0.05 were considered statistically significant.

## Supplementary information

Supplementary information

## References

[1] Ferlay J (2015). Cancer incidence and mortality worldwide: sources, methods and major patterns in GLOBOCAN 2012. Int. J. Cancer.

[2] Makarova-Rusher OV (2016). Population attributable fractions of risk factors for hepatocellular carcinoma in the United States. Cancer.

[3] Shin S (2016). Genetic lineage tracing analysis of the cell of origin of hepatotoxin-induced liver tumors in mice. Hepatology.

[4] Aloia L, McKie MA, Huch M (2016). Cellular plasticity in the adult liver and stomach. J Physiol.

[5] Kopp JL, Grompe M, Sander M (2016). Stem cells versus plasticity in liver and pancreas regeneration. Nat. Cell Biol..

[6] Merrell AJ, Stanger BZ (2016). Adult cell plasticity in vivo: de-differentiation and transdifferentiation are back in style. Nat. Rev. Mol. Cell Biol..

[7] Yimlamai D (2014). Hippo pathway activity influences liver cell fate. Cell.

[8] Schaub JR, Malato Y, Gormond C, Willenbring H (2014). Evidence against a stem cell origin of new hepatocytes in a common mouse model of chronic liver injury. Cell reports.

[9] Tarlow BD (2014). Bipotential adult liver progenitors are derived from chronically injured mature hepatocytes. Cell Stem Cell.

[10] Yanger K (2014). Adult hepatocytes are generated by self-duplication rather than stem cell differentiation. Cell Stem Cell.

[11] Ali N (2011). Hepatitis C virus-induced cancer stem cell-like signatures in cell culture and murine tumor xenografts. J. Virol..

[12] Westphalen CB (2014). Long-lived intestinal tuft cells serve as colon cancer-initiating cells. J. Clin. Investig..

[13] Nakanishi Y (2012). Dclk1 distinguishes between tumor and normal stem cells in the intestine. Nat. Genet..

[14] Fan M, Qian N, Dai G (2017). Expression and prognostic significance of doublecortin-like kinase 1 in patients with hepatocellular carcinoma. Oncol. Lett.

[15] Wang W, Zhang H, Wang L, Zhang S, Tang M (2016). miR-613 inhibits the growth and invasiveness of human hepatocellular carcinoma via targeting DCLK1. Biochem Biophys Res Commun.

[16] Ali N (2015). Inflammatory and oncogenic roles of a tumor stem cell marker doublecortin-like kinase (DCLK1) in virus-induced chronic liver diseases. Oncotarget.

[17] Ali N (2013). Fluvastatin interferes with hepatitis C virus replication via microtubule bundling and a doublecortin-like kinase-mediated mechanism. PLoS ONE.

[18] Sureban SM (2015). Plasma DCLK1 is a marker of hepatocellular carcinoma (HCC): Targeting DCLK1 prevents HCC tumor xenograft growth via a microRNA-dependent mechanism. Oncotarget.

[19] Nguyen CB (2016). (Z)-3,5,4'-Trimethoxystilbene Limits Hepatitis C and Cancer Pathophysiology by Blocking Microtubule Dynamics and Cell-Cycle Progression. Cancer Res.

[20] May R*, et al.* Dclk1 deletion in tuft cells results in impaired epithelial repair after radiation injury. *Stem cells*, (2013). 10.1002/stem.1566 PMC4603545 24123696

[21] Qu D (2015). Ablation of Doublecortin-Like Kinase 1 in the Colonic Epithelium Exacerbates Dextran Sulfate Sodium-Induced Colitis. PLoS ONE.

[22] Chandrakesan P (2014). DCLK1 facilitates intestinal tumor growth via enhancing pluripotency and epithelial mesenchymal transition. Oncotarget.

[23] Sureban SM (2014). XMD8-92 inhibits pancreatic tumor xenograft growth via a DCLK1-dependent mechanism. Cancer Lett..

[24] Sureban SM (2013). DCLK1 regulates pluripotency and angiogenic factors via microRNA-dependent mechanisms in pancreatic cancer. PLoS ONE.

[25] Lin PT, Gleeson JG, Corbo JC, Flanagan L, Walsh CA (2000). DCAMKL1 encodes a protein kinase with homology to doublecortin that regulates microtubule polymerization. J Neurosci.

[26] Shu T (2006). Doublecortin-like kinase controls neurogenesis by regulating mitotic spindles and M phase progression. Neuron.

[27] Monga SP (2015). beta-Catenin Signaling and Roles in Liver Homeostasis, Injury, and Tumorigenesis. Gastroenterology.

[28] Chen J, Rajasekaran M, Hui KM (2017). Atypical regulators of Wnt/beta-catenin signaling as potential therapeutic targets in Hepatocellular Carcinoma. Exp. Biol. Med. (Maywood).

[29] Souza GR (2010). Three-dimensional tissue culture based on magnetic cell levitation. Nat. Nanotechnol..

[30] Nusse R, Clevers H (2017). Wnt/beta-catenin signaling, disease, and emerging therapeutic modalities. Cell.

[31] van Noort M, Meeldijk J, van der Zee R, Destree O, Clevers H (2002). Wnt signaling controls the phosphorylation status of beta-catenin. J. Biol. Chem..

[32] Goretsky T (2018). Beta-catenin cleavage enhances transcriptional activation. Sci. Rep..

[33] Xu W, Kimelman D (2007). Mechanistic insights from structural studies of beta-catenin and its binding partners. J. Cell Sci..

[34] Grimes CA, Jope RS (2001). The multifaceted roles of glycogen synthase kinase 3beta in cellular signaling. Prog. Neurobiol..

[35] Selimovic D (2013). Bortezomib/proteasome inhibitor triggers both apoptosis and autophagy-dependent pathways in melanoma cells. Cell Signal..

[36] David JM, Rajasekaran AK (2012). Dishonorable discharge: the oncogenic roles of cleaved E-cadherin fragments. Cancer Res..

[37] Maretzky T (2005). ADAM10 mediates E-cadherin shedding and regulates epithelial cell-cell adhesion, migration, and beta-catenin translocation. Proc. Natl. Acad. Sci. USA.

[38] Marambaud P (2002). A presenilin-1/gamma-secretase cleavage releases the E-cadherin intracellular domain and regulates disassembly of adherens junctions. EMBO J..

[39] Azuma H (2007). Robust expansion of human hepatocytes in Fah-/-/Rag2-/-/Il2rg-/- mice. Nat. Biotechnol..

[40] Tummala KS (2017). Hepatocellular carcinomas originate predominantly from hepatocytes and benign lesions from hepatic progenitor cells. Cell Rep..

[41] Mu X (2015). Hepatocellular carcinoma originates from hepatocytes and not from the progenitor/biliary compartment. J. Clin. Investig..

[42] Han X (2019). Lineage tracing reveals the bipotency of SOX9(+) hepatocytes during liver regeneration. Stem Cell Rep..

[43] Deane NG (2001). Hepatocellular carcinoma results from chronic cyclin D1 overexpression in transgenic mice. Cancer Res.

[44] Nishida N (1994). Amplification and overexpression of the cyclin D1 gene in aggressive human hepatocellular carcinoma. Cancer Res..

[45] Wang W (2016). Blocking Wnt secretion reduces growth of hepatocellular carcinoma cell lines mostly independent of beta-catenin signaling. Neoplasia.

[46] Huber AH, Nelson WJ, Weis WI (1997). Three-dimensional structure of the armadillo repeat region of beta-catenin. Cell.

[47] Hughes CS, Postovit LM, Lajoie GA (2010). Matrigel: a complex protein mixture required for optimal growth of cell culture. Proteomics.

[48] Bissig KD (2010). Human liver chimeric mice provide a model for hepatitis B and C virus infection and treatment. J. Clin. Investig..

[49] Lipka J, Kapitein LC, Jaworski J, Hoogenraad CC (2016). Microtubule-binding protein doublecortin-like kinase 1 (DCLK1) guides kinesin-3-mediated cargo transport to dendrites. EMBO J..

[50] Nguyen CB, Houchen CW, Ali N (2017). APSA awardee submission: Tumor/cancer stem cell marker doublecortin-like kinase 1 in liver diseases. Exp. Biol. Med. (Maywood).

[51] Tiscornia G, Singer O, Verma IM (2006). Production and purification of lentiviral vectors. Nat Protoc.

