# Supplementary material for: Doublecortin-like kinase 1 promotes hepatocyte clonogenicity and oncogenic programming via non-canonical β-catenin-dependent mechanism
Source: Sci Rep. 2020 Jun 29;10:10578. doi: 10.1038/s41598-020-67401-y (PMC7324569; doi:10.1038/s41598-020-67401-y)
Supplement: Supplementary file 1 — Supplementary information [file 41598_2020_67401_MOESM1_ESM.pdf]

Doublecortin-like Kinase 1 Promotes Hepatocyte Clonogenicity and Oncogenic Programming via Non-Canonical β-Catenin-Dependent Mechanism

Running Title: DCLK1-induced β-catenin signaling in liver cancer

Naushad Ali[1,2,3,*], Charles B. Nguyen[1], Parthasarathy Chandrakesan[1,2,3], Roman F. Wolf[3], Dongfeng Qu[1,2], Randal May[1], Tatiana Goretsky[5], Javid Fazili[1,3], Terrence A. Barrett[5], Min Li[1,2], Mark Huycke[2,4], Michael S. Bronze[1], Courtney W. Houchen[1,2,3, *]

[1]Department of Medicine, Section of Digestive Diseases and Nutrition,
[2]Peggy and Charles Stephenson Cancer Center,
[3]Department of Veterans Affairs Medical Center,
[4]Department of Radiation Oncology
University of Oklahoma Health Sciences Center,
Oklahoma City, OK73104.

[5]Department of Internal Medicine, Division of Gastroenterology, University of Kentucky, Lexington, KY 40536.

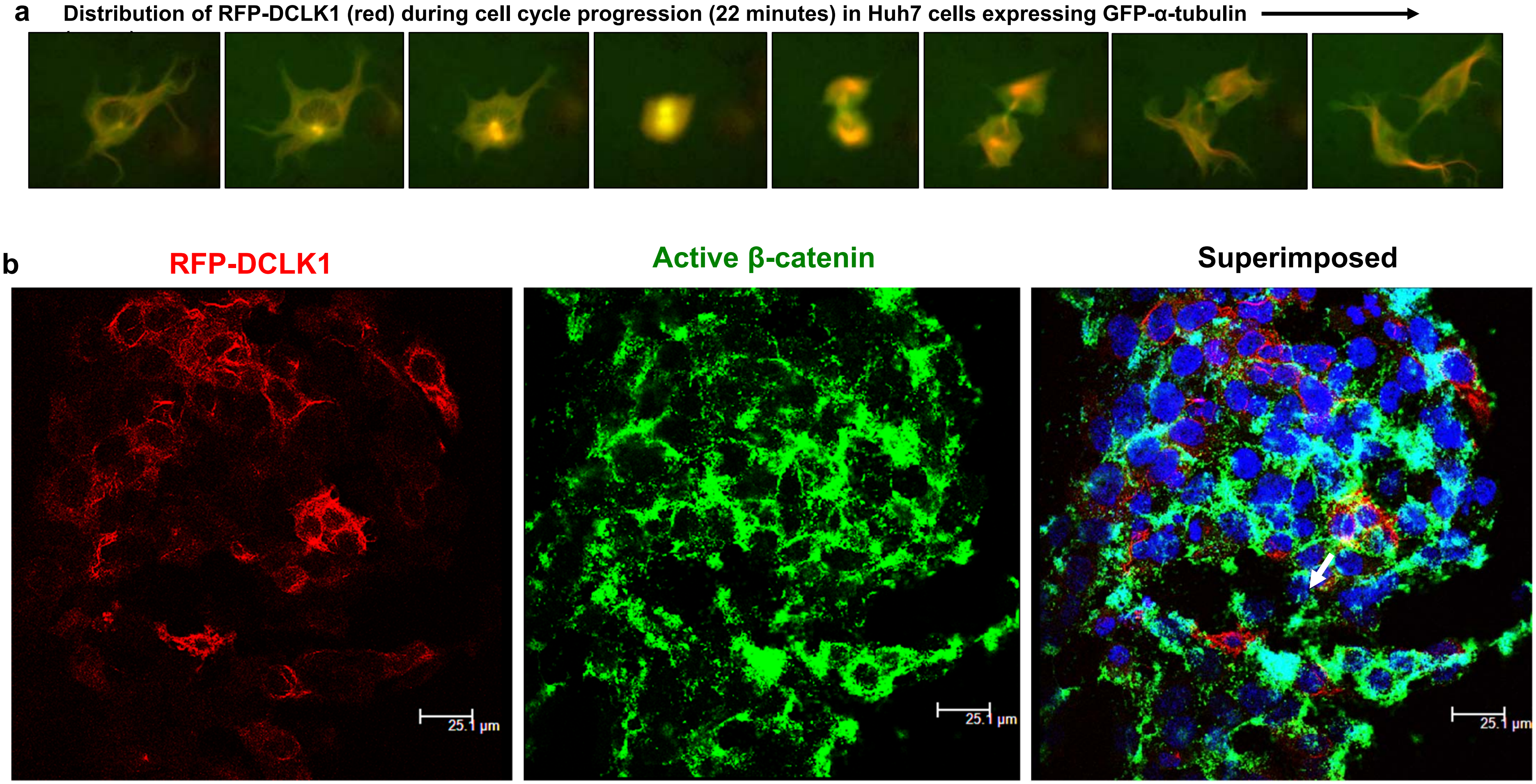

Supplementary Fig. S1

Dynamic distribution of recombinant RFP-DCLK1 in Huh7 cells during cell division and active β-catenin expression pattern in a colony of Huh7-RFP-DCLK1+ cells. **a** Live cell imaging of Huh7 cells expressing RFP-DCLK1 (Huh7-RFP-DCLK1, red) and GFP-tagged α-tubulin (green, showing microtubules). Yellow appearance reflects co- distribution/localization of RFP-DCLK1 with microtubules in a mitotic cell. **b** Confocal microscopy of Huh7-RFP-DCLK1 colony in monolayer culture. Green, active β-catenin stained with anti-ABC antibody; Blue, Dapi for nuclear stain.

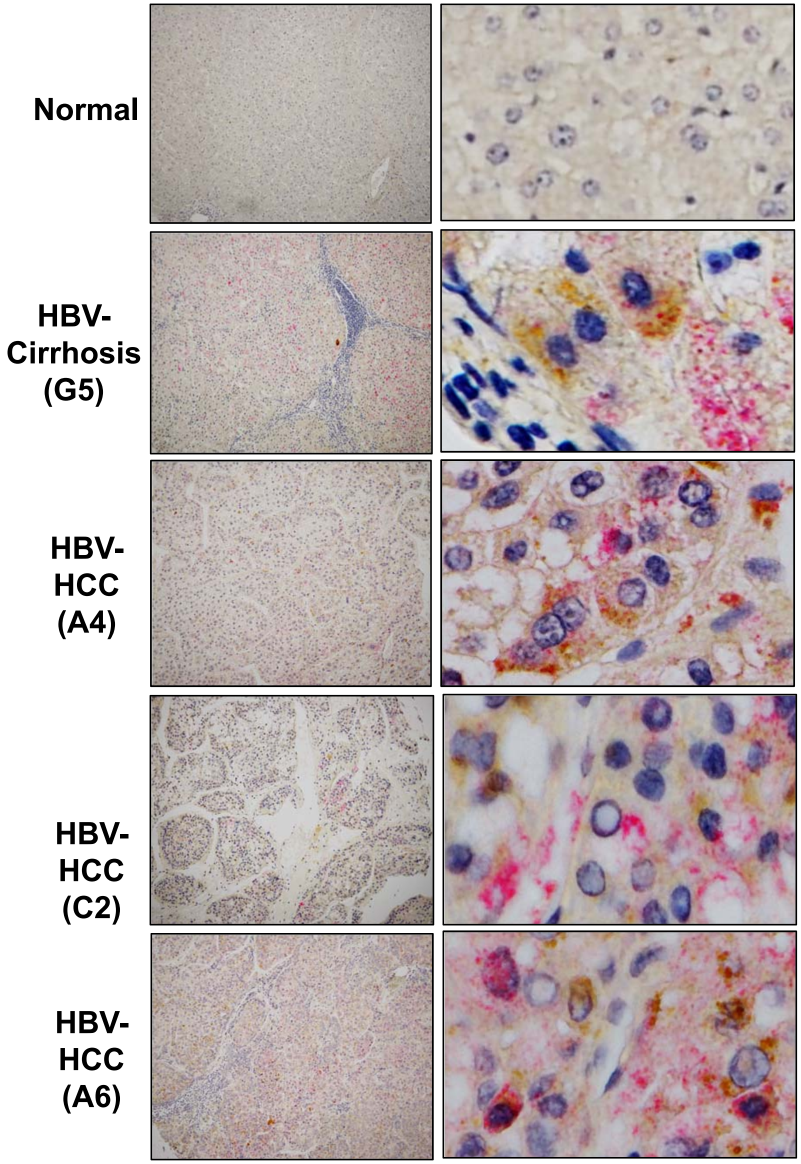

Supplementary Fig. S2
Livers of HBV patients with cirrhosis and HCC co-stained for DCLK1 and active β-catenin. Immunohistochemical staining of liver tissues (normal, cirrhosis and HCC) was simultaneously carried out with anti-ABC 8E7 mAb and anti-DCLK1 ab10902 antibodies. The co-staining of DCLK1 (brown) and active β-catenin (red) (10X image) in the same epithelial cells are highlighted in the right panel.

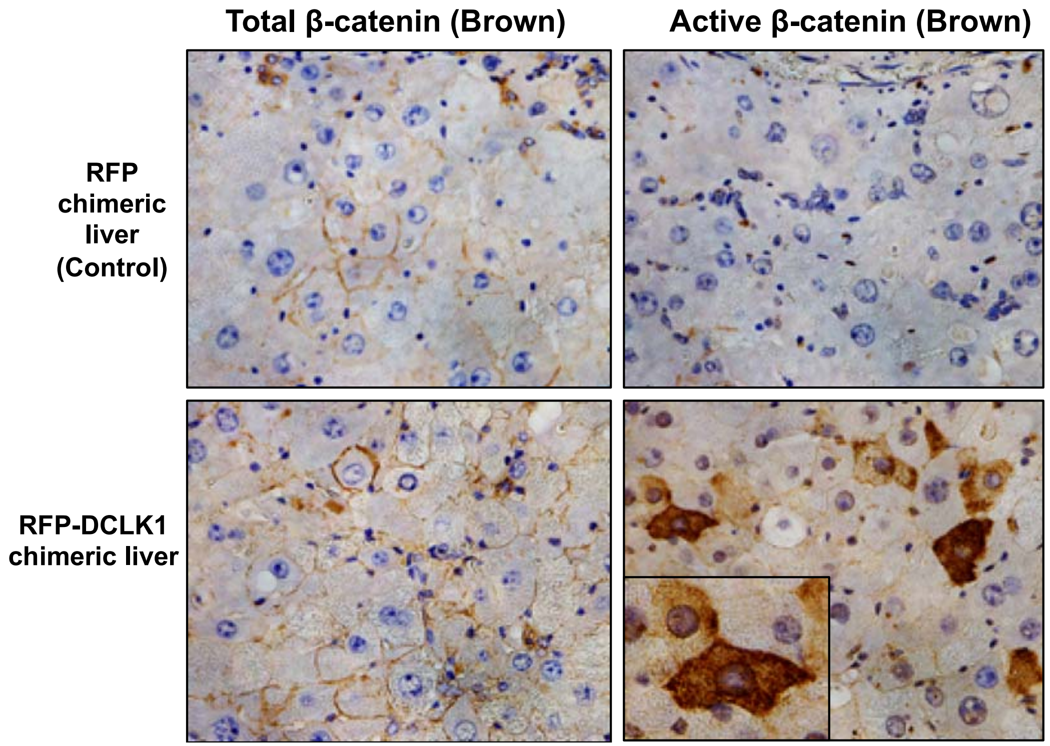

Supplementary Fig. S3.
Staining pattern of total and active β-catenin in the chimeric FRG livers. Extensive nucleo-cytoplasmic staining of active β-catenin (lower right panel) only in RFP-DCLK1 chimeric liver, (highlighted in the inset) and a typical membranous staining for total β-catenin (left panel) are shown.
